# Safety and efficacy of fecal microbiota transplantation for viral diseases: A systematic review of clinical trials

**DOI:** 10.1371/journal.pone.0311731

**Published:** 2024-10-21

**Authors:** Rasoul Ebrahimi, Mohammad Mahdi Masouri, Amir Abbas Salehi Amniyeh Khozani, Dana Ramadhan Hussein, Seyed Aria Nejadghaderi

**Affiliations:** 1 School of Medicine, Shahid Beheshti University of Medical Sciences, Tehran, Iran; 2 HIV/STI Surveillance Research Center, and WHO Collaborating Center for HIV Surveillance, Institute for Futures Studies in Health, Kerman University of Medical Sciences, Kerman, Iran; 3 Systematic Review and Meta-analysis Expert Group (SRMEG), Universal Scientific Education and Research Network (USERN), Tehran, Iran; NYU Grossman Long Island School of Medicine, UNITED STATES OF AMERICA

## Abstract

**Background:**

Gut microbiota play important roles in several diseases like viral infections. In this systematic review, our objective was to assess the efficacy and safety of fecal microbiota transplantation (FMT) in treating various viral diseases.

**Methods:**

We conducted searches on databases including PubMed, Web of Science, Scopus, and Google Scholar until November 2023. Clinical trials reported outcomes related to safety of FMT or its efficacy in patients with viral diseases were included. We excluded other types of studies that enrolled healthy individuals or patients with other disorders and did not use FMT. The assessment of bias risk was conducted using the National Institutes of Health (NIH) study quality evaluation tool.

**Results:**

Eight studies with total 196 participants were included. Viral diseases were human immunodeficiency virus (HIV), hepatitis B, COVID-19 and Clostridioides difficile coinfection, and cytomegalovirus colitis. In hepatitis B cases, HBeAg clearance was significant in those received FMT (p<0.01), while it was not significant in another one (p = 0.19). A clinical response was noted in 37.5% of patients with cytomegalovirus colitis, with an equal percentage achieving clinical remission post-FMT. There was a significant reduction in Clostridioides difficile relapse rate in FMT group than controls in coinfection of Clostridioides difficile and COVID-19 (2.17% vs. 42.5%, p<0.05). In patients with HIV, partial engraftment of the donor microbiome and increases in alpha diversity were observed after FMT. No severe adverse events were reported. Most studies had fair or good qualities.

**Conclusions:**

Our findings revealed FMT as a promising, safe treatment for some viral diseases. It improved viral clearance, clinical outcomes, and inflammation. However, the varying responses and small sample sizes call for more trials on FMT in viral diseases.

## Introduction

The digestive system has a diverse population of different microorganisms such as bacteria, fungi, viruses, and protozoa, overall make gut microbiota [1]. Currently, there is strong evidence supporting microbiota in regulating the balance between gut health and inflammation [2]. The occurrence of diverse diseases, including viral infections, has been associated with imbalances in gut microbiota [3].

Various factors, encompassing inherent factors like genetics and the aging process, as well as external factors such as medications, diet, and infections, can influence gut microbiota composition [4]. Probiotic and commensal organisms can lead to bolstering the immune system [5]. Antiviral mechanisms involve the release of antiviral peptides, modulation of leukocyte activity, improved integrity of the mucosal barrier, and prevention of viral attachment to cells [6]. Viral infections can disturb the symbiotic relationship between gut microbiota and mucosal immune mechanism [7]. This disruption can lead to gut microbiota dysbiosis, which inhibits the immune response and worsens viral diseases.

Strategies like fecal microbiota transplantation (FMT) and probiotic interventions are employed to rectify the imbalances in the gut microbiota [8]. FMT involves transferring the microbiota from a healthy donor to the recipient [9]. FMT was first used in China and due to the emergence of antibiotic resistance, scientists have re-evaluated its role in modern medicine [10, 11]. It has demonstrated better outcomes compared to antibiotic therapies when treating refractory and recurring cases of Clostridioides difficile (C. difficile) infection [12]. As a result, many research studies are exploring the potential advantages of FMT in treating non-communicable diseases like neuropsychiatric disorders, metabolic conditions, cancers, and inflammatory bowel disease (IBD) [13–17].

Several viruses, such as human immunodeficiency virus (HIV), parvoviruses, enteroviruses, and avian influenza virus, have been observed to disrupt the diversity of the gut microbiota in humans [18]. So, FMT is currently under investigation as a potential treatment for some viral infections like HIV [19]. Previous studies were conducted about the roles of FMT for patients with HIV infection [20, 21]. Recently, a secondary study evaluated efficacy and adverse events of FMT for HIV [20]. However, it included and pooled different types of observational and interventional studies, as well as case reports [20]. In addition, it did not evaluate the effects of FMT for other viral diseases. So, we systematically reviewed clinical trials to assess the efficacy and safety of FMT for various viral diseases.

## Methods

We undertook this systematic review adhering to the principles outlined in the PRISMA 2020 guidelines [22].

### Search strategy

We conducted searches on the PubMed, Web of Science, and Scopus databases up to November 2, 2023, with no limitations on language, date, or article type. We combined terms related to "Fecal microbiota transplantation" and "Viral diseases": ((("Feces" OR "fecal" OR "stool") AND ("microbiota" OR "Microbiome" OR "flora") AND ("Transplantation" OR "transfusion" OR "implant" OR "bacteriotherapy")) OR ("Donor Feces Infusion" OR "Intestinal Microbiome Transplant" OR "Intestinal Microbiota Transfer")) AND ("Viral Infection" OR "viral disease" OR "COVID-19" OR "HIV" OR "CMV" OR "Hepatitis" OR "HPV" OR "Herpes Simplex" OR "EBV" OR "VZ Virus") (S1 Table). We also investigated the Google Scholar search engine, as grey literature search following the completion of the full-text review [23]. Additionally, we carried out backward and forward citation searches for the included studies.

### Study selection

The records were imported to EndNote software and any duplicated items were removed using that. We included the followings: 1) clinical trial (with or without control arm) in any phase; 2) participants with any types of viral diseases (e.g., HIV, COVID-19, viral hepatitis, and cytomegalovirus infection); 3) the intervention had to be FMT; 4) in case of any control arms, placebo, standard treatment, or other modalities except for FMT should be used; and 5) at least outcomes related to the efficacy or safety should be reported. We excluded studies enrolled healthy participants or patients with disorders other than viral diseases, studies that did not use FMT as an intervention, and studies other than clinical trials (e.g., animal studies, in vitro studies, editorials, letters, perspectives, opinions, reviews, news, and books). Also, studies that evaluated gut microbiota patterns without using FMT were excluded.

Two reviewers (MMM and AASAK) independently evaluated the titles/abstracts of each study. Then, the same two reviewers independently reviewed full-texts of articles included from the previous step. Any disagreements were brought to the deliberation by discussion or consultation with the senior investigator (SAN).

### Data extraction

A data extraction sheet was designed by the senior investigator (SAN) in Microsoft Excel Office, version 2016. Then, two authors (RE and DRH) obtained the relevant data from the eligible studies independently. The principal investigator resolved discrepancies. The extracted variables were divided into four categories: 1) Study characteristics (first author’s name, year, country, and clinical trial phase and blinding); 2) Patient characteristics (age, sex, follow-up duration, type of viral disease, and underlying diseases); 3) Intervention characteristics (type, route of administration, schedule, and dose); and 4) Outcomes (effect sizes for each efficacy and safety outcome assessed in the intervention and control arms). In this systematic review, no missing data were encountered, and all relevant information from the included studies was fully available. Since this review did not involve a meta-analysis, there was no need for handling missing data or imputing values. All studies provided sufficient data to address the research questions and were included without the need for exclusions due to incomplete information.

### Quality assessment

Two authors (RE and DRH) individually evaluated the quality of each included article, and any disagreements were resolved through discussion. The National Institutes of Health (NIH) quality assessment tool for case series and controlled intervention studies was employed in this process [24]. In brief, the nine items for uncontrolled studies are the clarity of the study question, description of the study population, consecutive case inclusion, comparability of subjects, clear intervention description, well-defined outcome measures, adequate follow-up duration, appropriate statistical methods, and a clear description of results. For controlled intervention studies, there are 14 domains which are study randomization, concealment of allocation of treatment, blinding status for participants, providers, and assessors of outcomes, baseline group similarity, drop-out rates, intervention adherence, avoidance of other interventions, the use of valid outcome measures, sample size sufficiency and pre-specification of outcomes and subgroups were considered. The overall quality was categorized as poor, fair, and good for scores 0–3, 4–6, and 7–9 for uncontrolled studies and for scores 0–6, 7–10, 11–14 for controlled studies, respectively.

## Results

### Study selection

**Fig 1:**
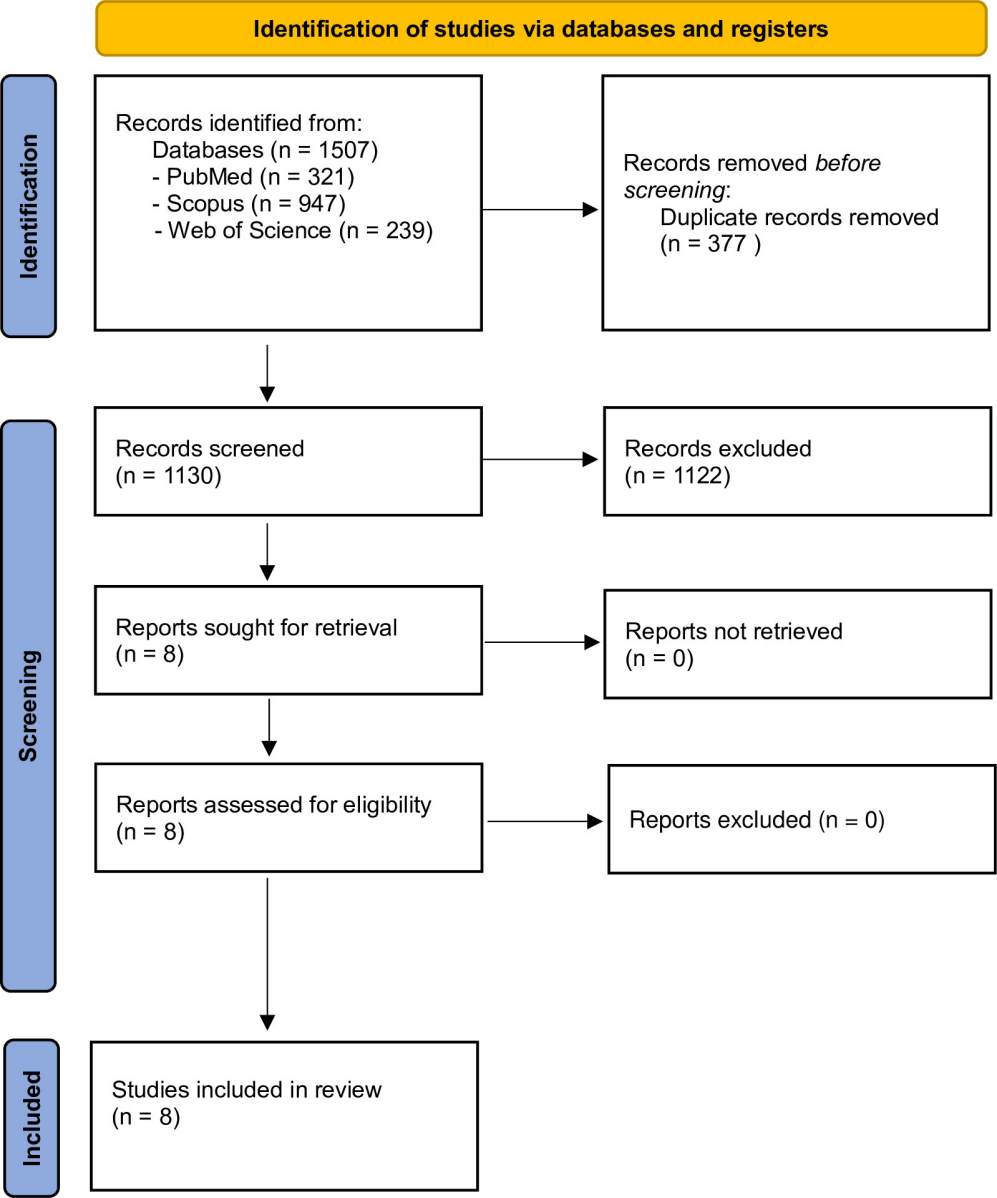
Study selection process.

The systematic search initially identified a total of 1507 articles. After removing duplicated results, 1130 studies underwent screening. In this step 1122 did not meet the eligibility criteria and were excluded. No studies were excluded in the full-text review. No other eligible studies were found in backward/forward citation searching and searching of Google Scholar. Finally, eight studies included in this systematic review [2, 25–31] (Fig 1).

### Characteristics of studies

Three studies were carried out in the United States [28–30], one study in each of the countries Poland [2], China [31], India [27], Romania [26], and Spain [25]. The studies were published between 2017 [29] and 2022 [26]. Among them, five studies were pilot trials [2, 25, 27, 29, 31]. The overall participant count was 196, and their ages ranged from 2 to 72 years. They were predominantly males (n = 136, 70%). Overall, there were 55 patients with HIV infection/acquired immunodeficiency syndrome (AIDS) [25, 28–30], 47 with chronic hepatitis B infection [27, 31], 86 with COVID-19 and C. difficile coinfection [26] and eight with cytomegalovirus colitis [2]. Out of these, 111 patients received FMT and the others were as control subjects. The duration of follow-up post-FMT ranged from eight [26] to 48 weeks [25] (Table 1).

**Table 1:** Study and patient characteristics.

Study ID	Country	NCT identifier	Design	Number of participants	Age (year)	Number of males	Duration of follow up	Type of viral disease and duration	Comorbidities	Objectives
SahBandar et al. 2020 [30]	USA	NA	Uncontrolled clinical trial	12 (Int: 12, Ctrl: 0)	Median (range): 59 (31–70)	12	24 weeks	HIV infection	None	Assessment of gut Fusobacteria abundance of HIV-infected individuals on cART Evaluation of expansion of TIGIT and PD-1 T cells and their cognate ligands in the periphery and GALT of virally suppressed HIV-infected individuals Evaluation of magnitude of peripheral anti-HIV CD8R T-cell responses to immune checkpoint blockade and associations with GALT immune phenotypes and gut microbial phyla abundance
Utay et al. 2020 [28]	USA	NCT03329560	Clinical trial	6 (Int: 6, Ctrl: 0)	Median (range): 39 (32–56)	6	20 weeks	HIV infection Median (range): 5.5 (2–35) years	None	Evaluation of Safety and Tolerability Changes in HIV Parameters Evaluation of microbiome Effects Changes in gene expression pathways Changes in markers of gut damage and inflammation Changes in anthropomorphics and hepatic steatosis
Karolewska-Bochenek et al. 2020 [2]	Poland	NA	Single-center, open, prospective trial	8 (Int: 8, Ctrl: 0)	Median (range): 15 (2–17)	4	6 weeks	Cytomegalovirus colitis	Ulcerative colitis Median (range): 15 (2–54) months	Changes in CMV tissue DNA PCR Evaluation of clinical response and clinical remission Changes in myo endoscopic scores Changes in laboratory markers (CRP, FCA) Adverse effects
Chauhan et al. 2020 [27]	India	NA	Non-randomized controlled pilot study	29^1^ (Int: 14, Ctrl: 15)	Int: 29 (25–35) Ctrl: 29 (24–38)	20 (Int: 9, Ctrl: 11)	6 months	Chronic hepatitis B (HBeAg positive)	None	Evaluation of HBeAg and HBsAg clearance Evaluation of HBV DNA and LFT Adverse events
Serrano-Villar et al. 2021 [25]	Spain	NCT03008941	Randomized, double-blind, placebo-controlled pilot trial	29 (Int 14; Ctrl: 15)	Mean (SD): 47 (11) Int: 48 (10) Ctrl: 46 (12)	25 (Int: 12, Ctrl: 13)	48 weeks	HIV infection	HCV positive patients: 6 in FMT arm & 3 in placebo arm	Changes in microbial alpha diversity Engraftment of donor’s microbiota on study participants Changes in microbiome taxonomic composition throughout the study Effects of repeated FMT of T-cells and plasma biomarkers Evaluation of safety data
Boicean et al. 2022 [26]	Romania	NA	Comparative retrospective single-center study​	86 (Int: 46, Ctrl: 40)	Mean: 67.65 Int: 67.63 Ctrl: 67.68	43 (Int: 23, Ctrl: 20)	8 weeks	COVID-19	Clostridioides difficile infection, cardiovascular disease, hypertension, neurological diseases, diabetes, obesity	Evaluating the role of FMT in decreasing the inflammatory syndrome, abdominal pain, and risk of recurrence
Ren et al. 2017 [31]	China	NA	Open-label pilot trial	18 (Int: 5, Ctrl: 13)	Median (range): 31 (19–68) Int: 27 (24–42) Ctrl: 33(19–68)	18 (Int: 5, Ctrl: 13)	Median (range) Int: 32 weeks (32–36) Ctrl: 32 weeks (28–40)	HBeAg-positive chronic hepatitis B	None	Changes in HBeAg and HBsAg titer Changes in ALT and HBV DNA Adverse events
Vujkovic-Cvijin et al. 2017 [29]	USA	NCT02256592	Clinical trial	8 (Int: 6, Ctrl: 2)	Median (range): 65 (31–72) Int: 65^2^ (31–72) Ctrl: 57 and 71	7 (Int: 6, Ctrl: 1)	24 weeks	HIV infection	None	Evaluation of microbiota shift in HIV infected FMT recipients toward donor profiles Changes in markers of HIV-associated inflammation

Abbreviations: USA: United States of America; NA: Not available; Int: intervention group; Ctrl: Control group; SD: standard deviation; HBV: Hepatitis B virus; HCV: Hepatitis C virus; IQR: Interquartile range; AIDS: acquired immunodeficiency syndrome; HIV: human immunodeficiency virus; ICB: immune checkpoint blockade; cART: combination antiretroviral therapy; FCA: feacal calprotectin; LFT: liver function test; ALT: alanine aminotransferase

1: There were two drop-outs and only 12 patients in the FMT arm completed the 6 sessions of FMT

2: The article mentioned a median age of 61 for the participants receiving FMT in the main text, while the median age for the FMT arm was 65 in its table.

Three studies administered FMT by colonoscopy [26, 29, 30], two via gastroscopy [27, 31], one by nasogastric tube [2], and two orally [25, 28]. FMT dosages and schedules varied. Some studies used 250 or 500 ml of stool suspensions introduced via colonoscopy [26, 30], while another study used capsules implying the delivery of 30 g of stool [25]. The frequency of administration varied from single [26] to multiple sessions [27], with intervals ranging from daily [2] to every four weeks [27, 31] (S2 Table).

### Efficacy

The detailed outcome results of each study are shown in Table 2.

**Table 2:** The outcome results of the studies.

Study ID	Viral response	Clinical outcomes	Markers of immunocompetence	Markers of inflammation and gut damage	Microbiota profile and engraftment	Anthropomorphics, hepatic steatosis and liver function tests
SahBandar et al. 2020 [30]	NA	NA	NA	NA	• No clear clustering between pre- and post-FMT microbial abundance was found. • Fusobacteria was not detected in the preparation of faecal bacteria from FMT HIV-uninfected donors. • Fusobacteria was detected in most of the HIV-infected recipients at entry (7/12), week 1 (5/12), week 2 (3/12), week 4 (6/12), week 8 (6/12), and week 24 (4/11). • Some FMT recipients retained detectable Fusobacteria while receiving antibiotic therapy post-FMT.	NA
Utay et al. 2020 [28]	HIV RNA Levels: • Two participants maintained <20 copies/mL at weeks 0, 6, and 26. • Other participants exhibited detectable HIV RNA levels.	NA	T Cell Counts and Ratios • No significant change in CD4+ T cell counts was observed between weeks 0 and 6 (496 [range 393–1029] vs 463 [range 329–900] cells/mm^3^; P = 0.56) or between weeks 6 and 26 (463 [range 329–900] vs 526 [range 329–1279] cells/mm^3^; P = 0.13). • CD8+ T cell counts tended to decrease between weeks 0 and 6 (845 [range 548–2587] vs 717 [range 512–2272] cells/mm3; P = 0.06) but did not change significantly between weeks 6 and 26 (717 [range 512–2272] vs 665 [range 558–3014] cells/mm3; P = 0.56). • No significant change in CD4/CD8 ratios was observed between weeks 0 and 6 (0.58 [range 0.40–0.89] vs 0.61 [range 0.40–0.98]; P = 0.31) or between weeks 6 and 26 (0.61 [range 0.40–0.98] vs 0.71 [range 0.42–0.96]; P = 0.22).	• Zonulin levels significantly increased from week 0 to week 6 (27.2 ng/mL to 31.7 ng/mL; P = 0.03). • No other biomarkers exhibited significant changes.	• Mean α diversity did not materially change between weeks 0 and 6 or between weeks 6 and 26. • α diversity increased in four participants (67%) from weeks 0 to 6, and at week 26, α diversity in three of the four participants was closer to that at week 0 than week 6. • Microbiome distribution shifted towards the donors’ distribution in three participants (50%) at week 6, but shifted away at week 26. • No significant changes were observed in any specific strains, species, genera, families, or phage distribution. • Seventeen strains, including several Bacteroides, Lachnospiraceae, and Ruminococcus strains, increased by >1% relative abundance. • Nineteen strains, including numerous oral microbes, decreased by >1% relative abundance.	Body Weight and Waist Circumference • No significant change in weight was observed between weeks 0 and 6 (median 0.5 kg; range -5 to 1.6; P = 0.75) or between weeks 6 and 26 (median 0.7 kg; range -0.5 to 1.4; P = 0.13). • Waist circumference changed by a median of -2.8 cm (range -5.0 to 2.0; P = 0.19) between weeks 0 and 6 and by -0.3 cm (range -4.0 to 3.3; P = 0.81) between weeks 6 and 26. Hepatic Steatosis • No significant change was observed in hepatic steatosis between weeks 0 and 26 based on transient elastography with controlled attenuation parameter values of 278 (range 212 to 345) and 276 (range 201 to 300) dB/m, (P = 0.91).
Karolewska-Bochenek et al. 2020 [2]	• After a 2-week course of FMT, seven out of eight patients became negative for CMV tissue DNA PCR. • In the remaining patient who did not respond to the two-week FMT course, a second FMT course was repeated, resulting in the eradication of CMV.	• At the 6th week following treatment with FMT, a clinical response was observed in three out of eight patients, and similarly, clinical remission was observed in three out of eight patients. • In one patient, a slight decrease in PUCAI score was observed after two courses of FMT. • An improvement in the Mayo endoscopic score was observed in six out of eight patients.	NA	• Two patients demonstrated normalization of CRP levels. • Three patients experienced a significant reduction in FCA levels.	NA	NA
Chauhan et al. 2020 [27]	• Two patients in FMT arm achieved HBeAg clearance in comparison to none in the AVT arm, 16.7% (2/12) versus 0/15 (P = 0.188). • Loss of HBeAg was seen in one patient after the first cycle of FMT, and in the second patient after 3 months. • No patient in either arm achieved HBsAg clearance. • A higher proportion of patients in the FMT arm experienced a decrease in HBV DNA levels than those in the AVT arm. • In the FMT arm, 25% (1/4) of patients who had positive HBV DNA at baseline became negative at 6 months. On the other hand, none of the two patients in the AVT arm who had positive HBV DNA at baseline became negative at 6 months.	NA	NA	NA	NA	• No significant change was observed in ALT levels between the FMT and AVT arms (− 6 IU/L in FMT arm and −1 IU/L in AVT arm, P = 0.683).
Serrano-Villar et al. 2021 [25]	NA	NA	• No trends were found in these parameters over time between the groups: Circulating CD4+ and CD8+ T cells, the CD4/CD8 ratio, markers of immune competence (CD4+ CD8+ T-cell count, CD4/CD8 ratio), immune activation (%HLADR+ CD38 on CD8+ T cells), senescence (CD28- and PD-1+ on CD8+ T cells), and plasma markers of inflammation and bacterial translocation.	• Compared to placebo, FMT caused a significant 0.5-fold-change decrease in IFABP levels. • The decrease in IFABP levels was observed at week 1 (FMT vs. placebo, p = 0.063) and reached statistical significance at week 4 (FMT vs. placebo, p = 0.040). • The decrease in IFABP levels remained stable until week 48 (FMT vs. placebo, p = 0.013).	• Bacterial richness increased incrementally in the FMT arm from 250 OTUs at week 0 to 287 at week 6, while in the placebo arm, it remained stable from 252 OTUs at week 0 to 254 at week 6 (differences between treatment arms from baseline through week 7, p = 0.014). • Long-lasting effects at this structural level were observed in the FMT arm (changes from baseline through week 6, p = 0.011 and from week 7 to week 48, p = 0.039). • Microbiome shifts were more pronounced in the FMT group than in the placebo group. • Engraftment of donor’s microbiota on FMT participants was more apparent in the four subjects who were exposed to antibiotics either before (n = 3) or immediately after the first FMT (n = 1) at both alpha and beta diversity levels. • The use of antibiotics after the baseline did not introduce drastic changes in the microbiota. • Several members of the Lachnospiraceae family, including Anaerostipes spp., Blautia spp., Dorea spp., and Fusicatenibacter spp., were significantly enriched over time in the FMT group. • Different members of the Ruminococcaceae family were also consistently detected across the study visits in the FMT arm.	NA
Boicean et al. 2022 [26]	NA	• FMT: 2.17% relapse rate (1 of 46 patients); Control: 42.5% relapse rate (17 of 40 patients); p < 0.05. • FMT: >91% no abdominal pain; Control: 82.5% persistent moderate pain; p < 0.05.	NA	CRP post-treatment (mean): 5.67 mg/L in the FMT group and 9.18 mg/l in the control group (p = 0.001). Leucocyte post-treatment (mean): 7695 cells/ml in the FMT group and 85,751 cells/mL in the control group (p = 0.001).	NA	NA
Ren et al. 2017 [31]	• HBeAg titer: Significant decline in FMT arm. Gradual decline after each FMT. Two participants achieved HBeAg clearance after one FMT, one after two FMTs. No clearance in control group (0/13) (P = 0.0001, Mantel-Cox’s test). • HBeAg seroconversion: No HBeAg seroconversion was observed at the end of follow-up. • HBV DNA: Detected at 4 weeks after each FMT and remained under lower limit of detection.	NA	NA	NA	NA	• ALT levels: Detected at 4 weeks after each FMT and remained under the lower limit of detection.
Vujkovic-Cvijin et al. 2017 [29]	NA	NA	NA	• Immune activation markers: No trends were evident in changes in the expression of the activation markers CD38 and HLADR on CD8C T cells, activity of the inflammation-associated indoleamine 2,3-dioxygenase pathway, or in plasma levels of the innate immune activation marker IL-6 or sCD14. • A nominal decrease in expression of the immune exhaustion-associated marker PD-1 on CD8C T cells was observed; though not statistically significant (P = 0.07, Benjamini-Hochberg false discovery rate Q = 0.29).	• FMT recipients’ fecal microbiota became significantly more similar to donor microbiota. • Most significant compositional relatedness at weeks 2 and 4 (P < 0.01), and less so at week 8 (P = 0.04). • Canberra β diversity distances shifted significantly toward donor profiles after FMT. • Proportions of shared OTUs between donors and recipients increased significantly after FMT (P = 0.0019). • Nominal increases in Faecalibacterium and Rikenellaceae, and decreases in Erysipelotrichaceae were observed in recipients post-FMT.	NA

Abbreviations: FCA: fecal calprotectin; ALT: Alanine aminotransferase; NA: Not available.

### Viral response

Two studies evaluated the HBeAg clearance [27, 31]. In the study by Chauhan et al., two (16.7%) patients in the FMT group had HBeAg clearance compared to none in the antiviral therapy (AVT) group (p = 0.19). Among these patients, HBsAg clearance was not observed in neither the FMT nor AVT arms [27]. Ren et al. reported HBeAg clearance in three participants in the FMT arm, while none of the participants in the control group achieved HBeAg clearance (p<0.01). Additionally, hepatitis B virus DNA levels were detected at four weeks after each FMT and remained under the lower limit of detection [31]. In the article by Utay et al., two participants maintained low HIV RNA levels throughout the study period, while others developed detectable HIV RNA levels [28]. Karolewska-Bochenek et al. reported that seven out of eight patients turned negative for cytomegalovirus (CMV) tissue DNA polymerase chain reaction (PCR) following a two-week FMT course and one patient who did not initially respond to FMT saw eradication of CMV after a repeated course of FMT [2].

### Clinical outcomes

Karolewska-Bochenek and colleagues observed a clinical response, defined as a reduction of the Pediatric Ulcerative Colitis Activity Index (PUCAI) score by at least 20 points, in 37.5% (three out of eight). Similarly, an equivalent percentage attained clinical remission (PUCAI score of less than 10 points). Additionally, an improvement in the Mayo endoscopic score was evident in 75% of the subjects (six out of eight) [2]. Boicean et al. showed that there was only one case of C. difficile infection (CDI) relapse (2.17% relapse rate). Among the 46 patients co-infected with SARS-CoV-2 and C. difficile who underwent FMT, there were only 17 cases of recurrence in the control group, resulting in a relapse rate of 42.5% (p<0.05) [26].

### Markers of immunocompetence

Utay et al. found no significant changes in CD4+ T cell counts between weeks 0 and 6 (p = 0.56), or from weeks 6 to 26 (p = 0.13). Likewise, there were no significant alterations in CD4+/CD8+ ratios during the periods of weeks 0 to 6 (p = 0.31) and weeks 6 to 26 (p = 0.22). Although CD8+ T cell counts exhibited a tendency to decrease from weeks 0 to 6 (p = 0.06), there was no significant change between weeks 6 and 26 (p = 0.56) [28]. Serrano-Villar and colleagues assessed circulating CD4+ and CD8+ T-cell counts, the CD4/CD8 ratio, immune activation markers (such as the percentage of HLADR+CD38 on CD8+ T cells), senescence markers (including CD28- and PD-1+ on CD8+ T cells), and plasma markers of inflammation and bacterial translocation. However, no discernible trends between groups over time were observed [25].

### Markers of inflammation and gut damage

Utay et al. noted only zonulin, a marker of gut permeability, increased significantly between weeks 0 and 6 (p = 0.03) [28]. Karolewska-Bochenek et al. reported fecal calprotectin significantly reduced in three patients and C-reactive protein (CRP) normalized in two patients six weeks after FMT [2]. Serrano-Villar et al. found no significant trends in plasma markers of inflammation and bacterial translocation between groups, although an early 0.5-fold-decline in the intestinal fatty-acid binding protein (IFABP) was noted in FMT group than controls (p = 0.06) which was statistically significant at week 4 (p = 0.04) and until week 48 (p = 0.01) [25]. Boicean et al. recorded a statistically significant improvement in CRP and white blood cell (WBC) count after FMT (p<0.05) [26]. While, Vujkovic-Cvijin et al. observed no significant changes in markers of HIV-associated inflammation following FMT (p = 0.07) [29].

### Microbiota profile and engraftment

SahBandar and colleagues emphasized variations in the relative abundances of all bacterial species from pre-fecal microbiota transplantation (FMT) to 24 weeks post-FMT. Principle component analysis did not show distinct clustering between microbial abundances before and after FMT. Interestingly, Fusobacteria, absent in the fecal bacteria preparation from FMT HIV-uninfected donors, was present in the majority of HIV-infected recipients. Additionally, some recipients of FMT who underwent post-treatment antibiotic therapy retained detectable Fusobacteria [30]. In the study by Utay et al., mean alpha diversity showed no significant change over weeks 0–6 (p = 0.29) and 6–26 (p = 0.21). Four participants (67%) saw an increase in alpha diversity initially, especially those with the lowest at week 0. By week 26, alpha diversity in three of these participants resembled week 0 more than week 6. At week 6, three participants (50%) showed a shift in their microbiome distribution towards the donors’ distribution but it was reversed by week 26. No significant changes were observed in specific microbial patterns or phage distribution. One long-term HIV patient experienced temporary improvement in constipation with notable microbiome shifts, but symptoms and microbial composition reverted by week 26 [28]. In the study by Vujkovic-Cvijin et al., FMT recipients showed a significant increase in similarity between their fecal microbiota and that of the donor, whereas control subjects did not show any significant change in their microbial compositional similarity to that of the donor microbial community. The compositional relatedness between donors and recipients were significant at weeks 2, 4 (p<0.01) and 8 (p = 0.04) following FMT. After FMT, there was increased proportions of shared operational taxonomic units between donor and recipient microbiota (p = 0.002), without any significant changes in specific microbial genera after adjusting for false discovery rates. However, the recipients showed an increasing presence of Faecalibacterium, Rikenellaceae, and a decrease in Erysipelotrichaceae post-FMT [29]. Serrano-Villar et al. discovered that FMT led to significant enhancements in alpha diversity when compared to individuals in the placebo arm up to week 6 (p = 0.01) and from weeks 7 to 48 (p = 0.04). Moreover, FMT induced a mild and temporary integration of the donor’s microbiota, with the potential for greater integration in cases with recent antibiotic use prior to FMT. The families Lachnospiraceae and Ruminococcaceae, which are typically diminished in HIV-infected patients, displayed more pronounced integration over time [25].

### Safety

Seven studies reported data regarding the adverse events [2, 25–29, 31]. None of the studies reported serious adverse events developed by the patients receiving FMT. However, the reported adverse events included mild abdominal pain [2, 28] and distension [25], nausea [2, 28], diarrhea and flatulence [25], and bloating [28]. According to Chauhan et al., only one patient experienced significant abdominal pain, requiring hospitalization [27]. In the study by Boicean et al., over 91% (42 out of 46) of FMT patients reported no abdominal pain post-treatment, versus 82.5% (33 out of 40) in the control group who experienced persistent moderate pain (p<0.05) [26] (Table 3).

**Table 3:** Reported adverse events among the included studies.

Study ID	Adverse events	Number of affected individuals	Severity	Onset
Serrano-Villar et al. 2021 [25]	Abdominal distension Flatulence Diarrhea	5 (35.7%)	Mild	Some hours after capsules intake until 48–72 h after
	No serious adverse events attributable to the intervention were reported			
	Overall, repeated oral capsular FMT from rationally selected donors was safe in PWH on ART			
Utay et al. 2020 [28]	Abdominal pain and nausea	1 (16.6%)	Grade 1	Abdominal pain at week 1 and nausea at week 2
	Bloating	1 (16.6%)	Mild to moderate	Mild bloating: 1 day each in weeks 1, 2, and 4 Moderate bloating: the last 2 days of week 6
	No other study drug-related adverse events were noted. Overall, FMT was safe and well-tolerated.			
Karolewska-Bochenek et al. 2020 [2]	Abdominal pain	5 (62.5%)	Mild	After the first dose of FMT
	Nausea	4 (50%)	NA	NA
	Vomiting	NA	NA	Within 2 hours after infusion
	Overall, no serious adverse effects were observed during and after infusions.			
Chauhan et al. 2020 [27]	Abdominal pain	2 (14.2%)	One of them had abdominal pain and required hospitalization for more than 24 h	NA
	Nausea	3 (21.4%)	NA	NA
	Vomiting	None	NA	NA
	Fever	None	NA	NA
	Diarrhea	None	NA	NA
	Bloating	3 (21.4%)	NA	NA
	Taste abnormality	None	NA	NA
	Retrosternal pain	1 (7.1%)	NA	NA
	42.8% (6/14) patients reported one minor adverse event.			
	Half (7/14) of the patients in FMT arm did not report any of the adverse events.			
Boicean et al. 2022 [26]	No reported severe adverse events during or after the procedure.			
Ren et al. 2017 [31]	No reported adverse events such as abdominal discomfort, diarrhea, and constipation.			
Vujkovic-Cvijin et al. 2017 [29]	No serious adverse events.			

Abbreviations: ART: Antiretroviral therapy; PWH: People with HIV; NA: Not available; FMT: fecal microbiota transplantation.

### Quality assessment

Uncontrolled studies met most of the quality criteria such as clear definition of study question, population, intervention, outcome measures, and results. With eight scores, they had good qualities. However, the consecutive case selection in the studies by Utay et al. and SahBandar et al. was not appropriate and in the study by Karolewska-Bochenek et al. the statistical methods were not described [2, 28, 30] (S3 Table).

The total scores of controlled interventional studies ranged from 7 to 13 (out of 14) with a mean score as 8.4. Studies by Boicean et al., Chauhan et al., Vujkovic-Cvijin et al. and Ren et al. had 7, 7, 7, and 8 scores respectively, indicating a fair quality [26, 27, 29, 31]. None of these studies received a score for randomization, treatment allocation concealment, and blinding of study participants and providers. The study by Serrano-Villar et al. received a score of 13 and met all of the assessed criteria except for reporting the sufficiency of the sample size [25] (S4 Table).

## Discussion

### Main findings

Our findings indicated that FMT had the potential to trigger clinical response, achieve remission, and eliminate CMV in individuals with CMV colitis. Furthermore, FMT led to a significantly lower relapse rate of CDI in patients co-infected with COVID-19 and C. difficile. HBeAg clearance was also achieved following FMT, even in patients with persistent HBeAg positivity after long-term AVT. FMT was found to increase zonulin levels and contribute to an early decline in IFABP, along with normalization of inflammatory markers in co-infected patients with COVID-19 and C. difficile. Additionally, partial engraftment of the donor microbiome and increases in alpha diversity were observed in participants after FMT. In terms of safety, there were no serious adverse effects.

### Clinical outcomes

FMT appears to be a viable and safe therapeutic choice for CMV colitis in pediatric patients with ulcerative colitis (UC). Similarly, Hsu et al. showed that clinical response one month after FMT was observed in 58.8% of pediatric IBD patients, with 64.7% achieving clinical remission and 44.1% attaining both clinical response and remission. The findings suggested that FMT exhibits enhanced safety and efficacy in the pediatric population when contrasted with the adult population [32]. In accordance with our results, Tun et al. found that FMT is an effective and safe therapy for pediatric and adolescent patients with CDI, demonstrating a pooled success rate of 86% (95% confidence interval (CI): 77–95%; p<0.001) across the overall cohort [33].

Additionally, FMT demonstrated potential in safely and efficiently treating CDI in patients with co-existing COVID-19, resulting a significantly lower CDI relapse rate. Similarly, another study noted that relapse occurred in only one individual five weeks post-FMT, while 98% of 54 participants exhibited clearance of C. difficile from their feces at 4–8 weeks after FMT, without any recurrences. There was a notable decrease in symptoms such as abdominal pain, rectal bleeding, and diarrhea. The outcomes support the idea of considering FMT as a preferable initial treatment for C. difficile, rather than delaying until multiple recurrences occur [34]. Furthermore, it has been demonstrated that FMT is more effective than vancomycin regimen for the treatment of recurrent CDI [35]. In this context, a clinical trial demonstrated that 81% of patients experiencing recurrent CDI achieved resolution of C. difficile-associated disease after a single session of FMT, in contrast to 31% of patients treated with vancomycin alone (p<0.001) [36]. In the same way with our findings, no recurrent C. difficile was observed during 16 months follow-up in all successfully treated patients, demonstrating an improvement of dysbiosis through FMT that prevents a repeated overgrowth of C. difficile [37]. Also, El-Salhy et al. evaluated the long-term effects of FMT in patients with irritable bowel syndrome (IBS). They included 77 patients with IBS who had responded to FMT and found that the patients had experienced improvement in abdominal symptoms and quality of life up to one year, as compared with three months following FMT. The patients had also comprehensive changes in the fecal bacterial profile and short-chain fatty acids [38].

### Viral response

FMT appears to have potential benefits for HBeAg clearance in chronic hepatitis B patients. Studies indicated that both spontaneous and treatment-induced HBeAg seroconversion were associated with decreased rates of disease progression towards cirrhosis and hepatocellular carcinoma, as well as improved survival rates [39, 40]. For patients with HBeAg-positive chronic hepatitis B, HBeAg seroconversion is a prerequisite for a definite course of AVT [41]. Despite many years of AVT with entecavir and tenofovir, only a small group of patients achieve HBeAg clearance or seroconversion. Accordingly, entecavir resulted in HBeAg loss in 53% of patients [42] and tenofovir treatment led to HBeAg loss in 49% and HBeAg seroconversion in 40% of patients five years after treatment [43]. Xie et al. found a gradual decline in serum HBsAg after each time of FMT in HBeAg negative chronic hepatitis B patients who remained HBsAg positive after more than one year of entecavir antiviral therapy, indicating that gut microbiota may be a new target for the treatment of HBeAg negative chronic hepatitis B [44].

FMT also seems to be an effective and safe treatment option for CMV colitis in patients with UC, as indicated by the eradication of CMV in the patients. During the last decade, the involvement of CMV in IBD flare-ups has been highly debated. While some authors advocate for an active role of CMV in inflammatory flares, others support the concept of the innocent bystander, suggesting that the virus does not contribute to the disease’s progression [45]. Although some studies indicated that AVT with ganciclovir or foscarnet improved outcomes in in UC flare with CMV infection [46–53], others found no benefits from these treatments [54, 55]. Given that both C. difficile and CMV infections can potentially impact the advancement of IBD, it is pertinent to examine studies investigating the application of FMT in C. difficile-infected patients with IBD. In the research conducted by Hourigan et al., all five participants (aged 10–17 years, four diagnosed with Crohn’s disease and one with UC) experiencing recurrent CDI witnessed the resolution of symptoms within three days following a single FMT session. Furthermore, 12–20 weeks after FMT, all patients tested negative for C. difficile toxin B PCR [56]. In the research conducted by Russel et al., FMT administration resulted in the clearance of CDI in two out of three children with both CDI and IBD [57]. The outcomes of these studies are encouraging, indicating that FMT, by restoring gut microbiota, may represent a highly promising treatment for infections arising due to gut dysbiosis. However, CMV and C. difficile are distinct pathogens and therefore, findings related to C. difficile cannot be directly applied to CMV.

### Markers of immunocompetence

We found that FMT did not modify CD4+ and CD8+ levels and CD4+/CD8+ ratio and there was also no change in the immune activation and senescence markers. While Serrano-Villar et al. chose three donors with comparable microbiota profiles to investigate diverse donor effects, the study by Utay et al. involved each recipient receiving FMT from a single donor. Additionally, the potential benefits might have been missed due to the small sample sizes, since Serrano-Villar et al. and Utay et al. performed FMT in 14 and six patients, respectively. Studies focusing on CD4+ T cell depletion and its direct impact on immune deficiency in HIV-infected patients mostly used peripheral blood samples [58–60]. Yet, it is in the gastrointestinal mucosa where the most significant and earliest reduction of CD4+ T cells is often observed, especially during the acute phase of HIV infection [61]. Given that an estimated 60% of CD4+ T cells are found in gut-associated lymphoid tissue and the observed incomplete restoration of these cell populations and intestinal microbial composition even with highly active antiretroviral therapy, the human intestine warrants significant focus in HIV research [62].

### Markers of inflammation and gut damage

Even with the use of antiretroviral therapy, individuals with HIV still face an elevated risk health complications, likely attributable to persistent inflammation [63]. An influential factor contributing to this inflammation is the heightened movement of microbial products through an permeable intestinal barrier [64]. Despite prolonged treatment, the levels of biomarkers indicating intestinal permeability, microbial translocation, and systemic inflammation continue to be elevated. Importantly, these biomarkers serve as predictive indicators for morbidity and mortality [65–67]. The elevated levels of zonulin in HIV infected patients may indicate more damage or turnover of tight junctions, potentially aligning with localized inflammation. On the other side, this rise could also suggest improved integrity of the gut barrier and reduced inhibition of zonulin production.

IFABP, a biomarker signifying intestinal damage, autonomously forecasts mortality in individuals undergoing HIV treatment [65, 67]. The early decline of IFABP among HIV infected patients points to FMT orally for acute HIV infection due to the disruption in gut immune system [68]. However, the absence of noticeable improvements in other biomarkers that represent various chronic inflammation pathways (sCD14, sCD164, sTNFR-II, IP-10, and D-dimer) suggests that the microbiome might not be directly connected with these inflammation pathways, or alternatively, more significant changes may be required to affect these biomarkers.

We found significant reduction of CRP, WBC count and fecal calprotectin following FMT. In the study by Wang et al., the serum concentrations of IL-1Ra, IL-6, Interferon gamma-induced protein 10 (IP-10), ENA-78, vascular cell adhesion molecule 1 (VCAM-1), and granulocyte colony-stimulating factor significantly decreased after FMT in patients with active UC (P<0.05). Furthermore, the serum concentrations of IL-6, IL-1Ra, IP-10, and VCAM-1 demonstrated significant positive correlations with both CRP and erythrocyte sedimentation rate [69]. Considering the pronounced inflammatory response in COVID-19 and CDI infections, with elevated levels of IL-6, TNF-α, IL-1β, and IP-10, as well as the reported dysbiosis [70–72], FMT could play a crucial role in the treatment of CDI or more severe conditions like COVID-19 and C. difficile co-infections.

### Microbiota profile and engraftment

In patients with HIV, there is greater abundance of Fusobacteria in subgingival plaques [73] and anal swabs [74] compared to HIV-uninfected group.

Previous studies proposed that incorporating dietary supplements containing probiotics [75] or undergoing FMT [76] could serve as a beneficial complementary approach to restore the gut microbial environment preceding immune checkpoint blockade. However, their data showed that FMT may be insufficient. We found that the gut colonization of Fusobacteria in HIV infection is persistent and may influence anti-HIV T-cell immunity to PD-1 or TIGIT blockade. Blocking these pathways may synergistically enhance the functions of HIV-specific CD8+ T cells [77].

Full engraftment of FMT was not observed, aligning with similar observations in conditions such as CDI and IBD. In these cases, FMT does not result in a complete substitution of the recipient’s microbiome with that of the donor. However, notable clinical improvements and alterations in the microbiome have been noted with FMT [78–80].

We found a modest shift towards donor microbiome profiles in treated HIV-infected patients whereas it was significantly smaller in magnitude than in patients with CDI, which may be due to the vastly decreased alpha diversity observed in patients with CDI. The concept of "resilience in diversity" suggests that a diverse microbial community has greater capacity to restore its composition after stress compared to a community with less diversity [81]. Therefore, the uniform and phylogenetically constrained community found in cases of recurrent CDI might be more prone to successful colonization by a diverse donor microbiome. Considering the effective engraftment observed in CDI, adopting a protocol that mimics C. difficile treatment, which include antibiotic conditioning prior to FMT is recommended.

### Safety

Overall, FMT was safe and well-tolerated in viral diseases that evaluated in our study. No serious adverse events were reported by the patients receiving FMT. However mild symptoms like abdominal pain, nausea, diarrhea, and flatulence were observed. FMT is also a safe, effective, and well-tolerated therapy for patients with IBD, as the safety analysis in the study by Tan et al. revealed that most adverse events following FMT were mild and self-resolving [82]. Furthermore, a one-year monitoring of individuals diagnosed with IBS who underwent FMT revealed that adverse effects such as abdominal pain, diarrhea, and constipation were mild and resolved on their own [83]. Lee et al. evaluated the long-term efficacy and safety of FMT for recurrent CDI. The study involved twenty-three patients who received FMT via a retention enema for recurrent CDI within the period from 2008 to 2012, and a follow-up questionnaire was completed by the patients four to eight years after the last FMT. They found that FMT produced no significant long-term adverse events, and about 30% of participants experienced improvements in pre-existing medical conditions, including IBD [84]. Also, Fang et al. conducted a study to assess the long-term safety and efficacy of monotherapy with a single fresh FMT for recurrent ulcerative colitis. The study sample was composed of 20 patients, with 10 participants in each the FMT group and the control group and the mean length of follow-up was 19.1 ± 10.1 months. They found that participants tolerated FMT treatment, and no adverse events were reported during the follow-up, except for one treatment-related Epstein–Barr virus infection which occurred within two weeks following FMT [85]. Marcella et al. conducted a review of 129 studies, including 4241 individuals, among whom the majority of patients had recurrent or refractory CDI, followed by UC, Crohn’s disease, and IBS. The commonly noted adverse effects associated with FMT included diarrhea (10%) and abdominal discomfort/pain/cramping (7%). Serious adverse events related to FMT, such as infections and fatalities, were documented in 1.4% of individuals who underwent the procedure [86]. Additionally, FMT has been documented as a safe procedure, with minimal reported adverse effects, even among patients with potential immunocompromise [87, 88].

### Methods of FMT administration

FMT can be administered via both the upper and lower gastrointestinal tract. Some research indicated that employing upper gastrointestinal administration necessitates careful consideration of potential adverse events, such as the occurrence of vomiting [89], and aspiration pneumonia [90]. Moreover, there are also risks when utilizing lower gastrointestinal tract administration methods. The advantages of colonoscopic administration include the ability to visually assess the colon, directly target specific areas, and deliver larger volumes of microbiota. Nevertheless, this approach carries risks associated with the use of anesthesia and the potential for bowel perforation [91]. Capsule-based FMT, on the other hand, has shown comparable effectiveness to colonoscopy in treating recurrent CDI and provides the benefits of convenience and patient satisfaction [92]. Besides efficacy, key factors such as patient comfort and compliance, cost-effectiveness, invasiveness, risk of aspiration and infection, the necessity for multiple drugs, and relapse rate inform physicians’ selection of the administration route [93].

### Strengths and limitations

To best of our knowledge, this is the first systematic review evaluating the efficacy and safety of FMT across various viral diseases. However, we had several limitations. The studies included in the review showed considerable variability in FMT protocols and dosages, reflecting diverse approaches across different research settings. Such variations could potentially influence the outcomes and comparability of the results. In addition, the number of participants involved in the studies was generally small which can significantly impact the generalizability and reliability of the findings, lead to reduced statistical power and make it difficult to detect differences between treatment and control groups. Furthermore, such limited samples may not represent the full range of patient diversity and heterogeneity, which may make the study findings less generalizable and applicable to patients with varying characteristics, comorbidities, or demographics. Moreover, a small sample size might not be capable of detecting subtle treatment differences or rare adverse events, which can make it challenging to evaluate the true efficacy and safety of the intervention. Additionally, the number of studies and patients involved in evaluating the effectiveness of FMT for a particular viral disease was also relatively low. Therefore, the results of our study should be interpreted with caution when it comes to drawing conclusions on the efficacy of FMT in treating specific viral infections. In addition, it is important to note that out of the 196 participants included in the study, 86 of them were diagnosed with COVID-19, which is a dominant viral infection. This means that the clinical course of COVID-19 may differ significantly from chronic virus infections such as chronic hepatitis B or CMV colitis. Therefore, findings of the study may not be generalizable across all types of viruses and viral infections. Also some studies lacked a control group, making it challenging to attribute the observed improvements solely to FMT. The age range of participants also varied, including both pediatric and adult populations, which adds another layer of complexity when interpreting the results and their applicability to broader patient demographics. These restrictions limited us about conducting meta-analysis to quantitatively report the pooled effect size. Moreover, reported adverse events can differ across studies, mainly due to differences in definitions and reporting methodologies. These disparities can make it challenging to compare results consistently across studies, leading to possible inconsistency in safety assessments. Also, the included studies mainly assessed the short-to-mid-term outcomes of FMT and the long-term effects of FMT on viral diseases and gut microbiota composition remain unclear.

## Conclusions

FMT may induce viral clearance, reduce inflammation, and improve clinical outcomes in specific viral diseases; however, the evidence remains limited, and the efficacy of FMT varies across different viral diseases. Caution is warranted for FMT in viral diseases due to varying responses and potential impacts on gut health. Further large-scale clinical trials assessing a personalized therapeutic management, is needed to establish the definite role of FMT in viral diseases and also a treatment protocol for practitioners. Additionally, studies with standardized adverse event reporting methodologies, clear definitions, well-defined and validated endpoints, larger sample sizes and longer follow-up periods are pivotal in providing more definitive conclusions regarding the safety and efficacy of FMT. Moreover, future studies should aim to investigate the differences in the efficacy of FMT protocols related to methods of administration, frequency of administrations, instilled fecal matter volume per administration, the use of adjuvant therapies to enhance the efficacy of FMT, and suitable donor details such as age, gender, diet, and lifestyle.

## Supporting information

S1 Table Search strategy for PubMed, Web of Science, Scopus, and Google Scholar. (DOCX)

S2 Table Intervention characteristics. (DOCX)

S3 Table Quality assessment of uncontrolled studies. (DOCX)

S4 Table Quality assessment of interventional controlled studies. (DOCX)

S1 Data Completed data extraction, FMT and Vir Dis. (XLSX)

S2 Data References FMT viral diseases 2. (XLSX)
