# Supplementary material for: Safety and efficacy of fecal microbiota transplantation for viral diseases: A systematic review of clinical trials
Source: PLoS One. 2024 Oct 21;19(10):e0311731. doi: 10.1371/journal.pone.0311731 (PMC11493255; doi:10.1371/journal.pone.0311731)
Supplement: S1 Table — (DOCX) [file pone.0311731.s001.docx]

# S1 Table. Search strategy for PubMed, Web of Science, Scopus, and Google Scholar.

| Database (search date) | Step | Search strategy | Number of results |
| --- | --- | --- | --- |
| PubMed  (November 2, 2023) | #1 | (((((Feces[MeSH Terms]) OR (Feces[Title/Abstract])) OR (faeces[Title/Abstract])) OR (faecal[Title/Abstract])) OR (fecal[Title/Abstract])) OR (stool[Title/Abstract]) | 217458 |
|  | #2 | ((((microbiota[MeSH Terms]) OR (Microbiota[Title/Abstract])) OR (Microbiome[Title/Abstract])) OR (flora[Title/Abstract])) OR (microflora[Title/Abstract]) | 189961 |
|  | #3 | (((((Transplantation[MeSH Terms]) OR (Transplant*[Title/Abstract])) OR (transfusion[Title/Abstract])) OR (implant*[Title/Abstract])) OR (instillation[Title/Abstract])) OR (bacteriotherapy[Title/Abstract]) | 1354650 |
|  | #4 | #1 AND #2 AND #3 | 6191 |
|  | #5 | ((((((Fecal Microbiota Transplantation[MeSH Terms]) OR (Fecal Microbiota Transplant*[Title/Abstract])) OR (Fecal Microbiome Transplant*[Title/Abstract])) OR (Fecal Transplant*[Title/Abstract])) OR (Donor Feces Infusion*[Title/Abstract])) OR (Intestinal Microbiome Transplant*[Title/Abstract])) OR (Intestinal Microbiota Transfer*[Title/Abstract]) | 5446 |
|  | #6 | #4 OR #5 | 6784 |
|  | #7 | (((((((((((((((((((((((((((((((((((((((((((((((((((((((((((((((((((((((((((((((((((((((((((((((((((((((((Virus Diseases[MeSH Terms]) OR (Virus Disease*[Title/Abstract])) OR (Viral Infection*[Title/Abstract])) OR (viral disease*[Title/Abstract])) OR (COVID-19[MeSH Terms])) OR (COVID-19[Title/Abstract])) OR (COVID 19[Title/Abstract])) OR (SARS-CoV-2[Title/Abstract])) OR (Coronavirus[Title/Abstract])) OR (2019-nCoV[Title/Abstract])) OR (Acquired Immunodeficiency Syndrome[MeSH Terms])) OR (Acquired Immunodeficiency Syndrome[Title/Abstract])) OR (AIDS[Title/Abstract])) OR (HIV[MeSH Terms])) OR (HIV[Title/Abstract])) OR (Human Immunodeficiency Virus[Title/Abstract])) OR (AIDS Virus[Title/Abstract])) OR (immunodeficiency associated virus[Title/Abstract])) OR (Lymphadenopathy Associated Virus[Title/Abstract])) OR (lymphadenopathy associated retrovirus[Title/Abstract])) OR (Human T Cell Lymphotropic Virus Type III[Title/Abstract])) OR (Human T Cell Leukemia Virus Type III[Title/Abstract])) OR (LAV-HTLV-III[Title/Abstract])) OR (Acquired Immune Deficiency Syndrome Virus[Title/Abstract])) OR (HTLV-III[Title/Abstract])) OR (Acquired Immunologic Deficiency Syndrome[Title/Abstract])) OR (LAV (AIDS[Title/Abstract]))) OR (Cytomegalovirus Infections[MeSH Terms])) OR (Cytomegalovirus[Title/Abstract])) OR (Cytomegalovirus Inclusion Disease[Title/Abstract])) OR (Inclusion Disease[Title/Abstract])) OR (CMV[Title/Abstract])) OR (CMV Inclusion*[Title/Abstract])) OR (Hepatitis[MeSH Terms])) OR (Hepatitis[Title/Abstract])) OR (Yellow Fever[Title/Abstract])) OR (Zika Virus[Title/Abstract])) OR (Zika Fever[Title/Abstract])) OR (ZikV Infection[Title/Abstract])) OR (Encephalitis, Viral[MeSH Terms])) OR (Encephalitis[Title/Abstract])) OR (Meningitis, Viral[MeSH Terms])) OR (Meningitis[Title/Abstract])) OR (Adenovirus[Title/Abstract])) OR (Herpesvirus[Title/Abstract])) OR (B Virus[Title/Abstract])) OR (Papillomavirus Infections[MeSH Terms])) OR (Papillomavirus[Title/Abstract])) OR (HPV[Title/Abstract])) OR (Parvoviridae Infections[MeSH Terms])) OR (Parvovirus[Title/Abstract])) OR (Polyomavirus Infections[MeSH Terms])) OR (Polyomavirus[Title/Abstract])) OR (Poxviridae Infections[MeSH Terms])) OR (Poxviridae[Title/Abstract])) OR (Poxvirus[Title/Abstract])) OR (Smallpox[Title/Abstract])) OR (Cowpox[Title/Abstract])) OR (Vaccinia[Title/Abstract])) OR (Conjunctivitis, Viral[MeSH Terms])) OR (Viral Conjunctivitis[Title/Abstract])) OR (Keratitis, Herpetic[MeSH Terms])) OR (Herpetic Keratitis[Title/Abstract])) OR (Herpes Simplex[Title/Abstract])) OR (Herpes Simplex[MeSH Terms])) OR (Warts[MeSH Terms])) OR (Wart*[Title/Abstract])) OR (Verruca[Title/Abstract])) OR (Epstein-Barr Virus Infections[MeSH Terms])) OR (Epstein Barr Virus[Title/Abstract])) OR (EBV[Title/Abstract])) OR (Dengue[MeSH Terms])) OR (Dengue[Title/Abstract])) OR (Breakbone Fever[Title/Abstract])) OR (Dengue Fever[Title/Abstract])) OR (Hemorrhagic Fevers, Viral[MeSH Terms])) OR (Hemorrhagic Fever[Title/Abstract])) OR (Influenza, Human[MeSH Terms])) OR (Influenza[Title/Abstract])) OR (Flu[Title/Abstract])) OR (Measles[MeSH Terms])) OR (Measles[Title/Abstract])) OR (Mumps[MeSH Terms])) OR (Mumps[Title/Abstract])) OR (Parotitis[Title/Abstract])) OR (Rubella[MeSH Terms])) OR (Rubella[Title/Abstract])) OR (Chickenpox[MeSH Terms])) OR (Chickenpox[Title/Abstract])) OR (Poliomyelitis[MeSH Terms])) OR (Poliomyelitis[Title/Abstract])) OR (Polio[Title/Abstract])) OR (Rabies[MeSH Terms])) OR (Rabies[Title/Abstract])) OR (Norovirus[MeSH Terms])) OR (Norovirus[Title/Abstract])) OR (Rotavirus[MeSH Terms])) OR (Rotavirus[Title/Abstract])) OR (Neonatal Calf Diarrhea Virus[Title/Abstract])) OR (Herpesvirus 3, Human[MeSH Terms])) OR (Varicella-Zoster virus[Title/Abstract])) OR (Varicella Zoster Virus[Title/Abstract])) OR (VZ Virus[Title/Abstract])) OR (Herpes zoster Virus[Title/Abstract])) OR (Varicella Zoster Virus Infection[MeSH Terms])) OR (Congenital Varicella Syndrome[Title/Abstract]) | 1978350 |
|  | #8 | #6 AND #7 | 321 |
| Web of Science  (November 2, 2023) | #1 | ((((AB=(Feces)) OR AB=(faeces)) OR AB=(faecal)) OR AB=(fecal)) OR AB=(stool) | 158558 |
|  | #2 | (((AB=(Microbiome)) OR AB=(microbiota)) OR AB=(flora)) OR AB=(microflora) | 164414 |
|  | #3 | ((((AB=(Transplant*)) OR AB=(transfusion)) OR AB=(implant*)) OR AB=(instillation)) OR AB=(bacteriotherapy) | 841094 |
|  | #4 | #1 AND #2 AND #3 | 4512 |
|  | #5 | (((((AB=("Fecal Microbiota Transplant*")) OR AB=("Fecal Microbiome Transplant*")) OR AB=("Fecal Transplant*")) OR AB=("Donor Feces Infusion*")) OR AB=("Intestinal Microbiome Transplant*")) OR AB=("Intestinal Microbiota Transfer*") | 3126 |
|  | #6 | #4 OR #5 | 4566 |
|  | #7 | ((((((((((((((((((((((((((((((((((((((((((((((((((((((((((((((((((((AB=("Virus Disease*")) OR AB=("Viral Infection*")) OR AB=("viral disease*")) OR AB=("COVID-19")) OR AB=("COVID 19")) OR AB=("SARS-CoV-2")) OR AB=(Coronavirus)) OR AB=("2019-nCoV")) OR AB=("Acquired Immunodeficiency Syndrome")) OR AB=(AIDS)) OR AB=(HIV)) OR AB=("Human Immunodeficiency Virus")) OR AB=("immunodeficiency associated virus")) OR AB=("Lymphadenopathy Associated Virus")) OR AB=("lymphadenopathy associated retrovirus")) OR AB=("Human T Cell Lymphotropic Virus Type III")) OR AB=("Human T Cell Leukemia Virus Type III")) OR AB=("LAV-HTLV-III")) OR AB=("Acquired Immune Deficiency Syndrome")) OR AB=("HTLV-III")) OR AB=("Acquired Immunologic Deficiency Syndrome")) OR AB=(Cytomegalovirus)) OR AB=("Inclusion Disease")) OR AB=(CMV)) OR AB=(Hepatitis)) OR AB=("Yellow Fever")) OR AB=("Zika Virus")) OR AB=("Zika Fever")) OR AB=("ZikV Infection")) OR AB=(Encephalitis)) OR AB=(Meningitis)) OR AB=(Adenovirus)) OR AB=(Herpesvirus)) OR AB=("B Virus")) OR AB=(Papillomavirus)) OR AB=(HPV)) OR AB=(Parvovirus)) OR AB=(Polyomavirus)) OR AB=(Poxvirus)) OR AB=(Smallpox)) OR AB=(Cowpox)) OR AB=(Vaccinia)) OR AB=("Viral Conjunctivitis")) OR AB=("Herpetic Keratitis")) OR AB=("Herpes Simplex")) OR AB=("Wart*")) OR AB=(Verruca)) OR AB=("Epstein-Barr Virus")) OR AB=("Epstein Barr Virus")) OR AB=(EBV)) OR AB=(Dengue)) OR AB=("Hemorrhagic Fever")) OR AB=(Influenza)) OR AB=(Flu)) OR AB=(Measles)) OR AB=(Mumps)) OR AB=(Parotitis)) OR AB=(Rubella)) OR AB=(Chickenpox)) OR AB=(Poliomyelitis)) OR AB=(Polio*)) OR AB=(Rabies)) OR AB=(Norovirus)) OR AB=(Rotavirus)) OR AB=("Neonatal Calf Diarrhea Virus")) OR AB=("Varicella-Zoster")) OR AB=("Varicella Zoster" )) OR AB=("Herpes zoster")) OR AB=("Congenital Varicella Syndrome") | 1453616 |
|  | #8 | #6 AND #7 | 239 |
| Scopus (November 2, 2023) | #1 | TITLE-ABS-KEY ( feces ) OR TITLE-ABS-KEY ( faeces ) OR TITLE-ABS-KEY ( faecal ) OR TITLE-ABS-KEY ( fecal ) OR TITLE-ABS-KEY ( stool ) | 322409 |
|  | #2 | TITLE-ABS-KEY ( microbiome ) OR TITLE-ABS-KEY ( microbiota ) OR TITLE-ABS-KEY ( flora ) OR TITLE-ABS-KEY ( microflora ) | 342420 |
|  | #3 | ( TITLE-ABS-KEY ( transplant* ) OR TITLE-ABS-KEY ( transfusion ) OR TITLE-ABS-KEY ( implant* ) OR TITLE-ABS-KEY ( instillation ) OR TITLE-ABS-KEY ( bacteriotherapy ) ) | 2126370 |
|  | #4 | #1 AND #2 AND #3 | 10569 |
|  | #5 | ( TITLE-ABS-KEY ( "Fecal Microbiota Transplant*" ) OR TITLE-ABS-KEY ( "Fecal Microbiome Transplant*" ) OR TITLE-ABS-KEY ( "Fecal Transplant*" ) OR TITLE-ABS-KEY ( "Donor Feces Infusion*" ) OR TITLE-ABS-KEY ( "Intestinal Microbiome Transplant*" ) OR TITLE-ABS-KEY ( "Intestinal Microbiota Transfer*" ) ) | 9582 |
|  | #6 | #4 OR #5 | 10674 |
|  | #7 | ( TITLE-ABS-KEY ( "Virus Disease*" ) OR TITLE-ABS-KEY ( "Viral Infection*" ) OR TITLE-ABS-KEY ( "viral disease*" ) OR TITLE-ABS-KEY ( "COVID-19" ) OR TITLE-ABS-KEY ( "COVID 19" ) OR TITLE-ABS-KEY ( "SARS-CoV-2" ) OR TITLE-ABS-KEY ( coronavirus ) OR TITLE-ABS-KEY ( "2019-nCoV" ) OR TITLE-ABS-KEY ( "Acquired Immunodeficiency Syndrome" ) OR TITLE-ABS-KEY ( aids ) OR TITLE-ABS-KEY ( hiv ) OR TITLE-ABS-KEY ( "Human Immunodeficiency Virus" ) OR TITLE-ABS-KEY ( "immunodeficiency associated virus" ) OR TITLE-ABS-KEY ( "lymphadenopathy associated retrovirus" ) OR TITLE-ABS-KEY ( "Human T Cell Lymphotropic Virus Type III" ) OR TITLE-ABS-KEY ( "Human T Cell Leukemia Virus Type III" ) OR TITLE-ABS-KEY ( "LAV-HTLV-III" ) OR TITLE-ABS-KEY ( "Acquired Immune Deficiency Syndrome" ) OR TITLE-ABS-KEY ( "HTLV-III" ) OR TITLE-ABS-KEY ( "Acquired Immunologic Deficiency Syndrome" ) OR TITLE-ABS-KEY ( cytomegalovirus ) OR TITLE-ABS-KEY ( "Inclusion Disease" ) OR TITLE-ABS-KEY ( cmv ) OR TITLE-ABS-KEY ( hepatitis ) OR TITLE-ABS-KEY ( "Yellow Fever" ) OR TITLE-ABS-KEY ( "Zika Virus" ) OR TITLE-ABS-KEY ( "Zika Fever" ) OR TITLE-ABS-KEY ( "ZikV Infection" ) OR TITLE-ABS-KEY ( encephalitis ) OR TITLE-ABS-KEY ( meningitis ) OR TITLE-ABS-KEY ( adenovirus ) OR TITLE-ABS-KEY ( herpesvirus ) OR TITLE-ABS-KEY ( "B Virus" ) OR TITLE-ABS-KEY ( papillomavirus ) OR TITLE-ABS-KEY ( hpv ) OR TITLE-ABS-KEY ( parvovirus ) OR TITLE-ABS-KEY ( polyomavirus ) OR TITLE-ABS-KEY ( poxvirus ) OR TITLE-ABS-KEY ( smallpox ) OR TITLE-ABS-KEY ( cowpox ) OR TITLE-ABS-KEY ( vaccinia ) OR TITLE-ABS-KEY ( "Viral Conjunctivitis" ) OR TITLE-ABS-KEY ( "Herpetic Keratitis" ) OR TITLE-ABS-KEY ( "Herpes Simplex" ) OR TITLE-ABS-KEY ( "Wart*" ) OR TITLE-ABS-KEY ( verruca ) OR TITLE-ABS-KEY ( "Epstein-Barr Virus" ) OR TITLE-ABS-KEY ( "Epstein Barr Virus" ) OR TITLE-ABS-KEY ( ebv ) OR TITLE-ABS-KEY ( dengue ) OR TITLE-ABS-KEY ( "Hemorrhagic Fever" ) OR TITLE-ABS-KEY ( influenza ) OR TITLE-ABS-KEY ( measles ) OR TITLE-ABS-KEY ( mumps ) OR TITLE-ABS-KEY ( parotitis ) OR TITLE-ABS-KEY ( rubella ) OR TITLE-ABS-KEY ( chickenpox ) OR TITLE-ABS-KEY ( poliomyelitis ) OR TITLE-ABS-KEY ( "Polio*" ) OR TITLE-ABS-KEY ( rabies ) OR TITLE-ABS-KEY ( norovirus ) OR TITLE-ABS-KEY ( rotavirus ) OR TITLE-ABS-KEY ( "Neonatal Calf Diarrhea Virus" ) OR TITLE-ABS-KEY ( "Varicella-Zoster" ) OR TITLE-ABS-KEY ( "Varicella Zoster" ) OR TITLE-ABS-KEY ( "Herpes zoster" ) OR TITLE-ABS-KEY ( "Congenital Varicella Syndrome" ) OR TITLE-ABS-KEY ( vzv ) OR TITLE-ABS-KEY ( flu ) ) | 2766287 |
|  | #8 | #6 AND #7 | 947 |
| Google Scholar  (November 13, 2023) | #1 | "Fecal Microbiota Transplantation" AND "Viral Disease" | 81 |
