# Supplementary material for: Safety and efficacy of fecal microbiota transplantation for viral diseases: A systematic review of clinical trials
Source: PLoS One. 2024 Oct 21;19(10):e0311731. doi: 10.1371/journal.pone.0311731 (PMC11493255; doi:10.1371/journal.pone.0311731)
Supplement: S2 Table — (DOCX) [file pone.0311731.s002.docx]

# S2 Table. Intervention characteristics.

| Study ID | Route of FMT administration | Schedule and dose of FMT administration | Other drugs in the intervention group | Type of control medication | Route of administration of control medication | Schedule and dose of control medication |
| --- | --- | --- | --- | --- | --- | --- |
| SahBandar et al. 2020 (1) | Colonoscopy | Participants underwent standard bowel purge (Golytely) the day preceding FMT and the 250 mL stool suspension was introduced via colonoscopy and delivered into the ileum, cecum, and ascending colon. | Stable combination ART - number of Abx+ patients* : 9 | None | None | None |
| Utay et al. 2020 (2) | Oral | Each participant received the investigational product (encapsulated lyophilized FMT) derived from 150 grams of stool (lyophilized to approximately 2.25 g) from one of three donors weekly for 6 weeks. | ART [median duration of usage was 5.5 (range 2-7 years) years] | None | None | None |
| Karolewska-Bochenek et al. 2020 (3) | NG tube | Every day during the whole induction FMT course, the study subject took a proton pump inhibitor at a dose 1 mg/kg and the anti-vomiting drug ondansetron 4–8 mg orally in the morning on the day of FMT administration. Each patient received 1 x 100 ml or 1 x 50 ml FMT for patients with body weight ≥40 kg and <40 kg, respectively, via NG tube, for 5 days in each of 2 weeks (in total, 10 FMT procedures in 12 days). FMT was administered in 10- min sessions. All the patients were monitored for 1 h and were allowed to drink 1 h and eat 2 h after FMT administration. | Glucocorticosteroids, Azathioprine, Aminosalicylates, Cyclosporine A, Infliximab | None | None | None |
| Chauhan et al. 2020 (4) | Gastroscope | Six sessions of FMT at 4 weeks intervals - For every FMT session, approximately 30 g of fresh stool, diluted in 150 ml of saline, was used. (Two drop outs : One patient received five cycles, and one patient received only one cycle) | AVT (Entecavir &/or tenofovir) - The number of patients being on dual therapy in FMT arm was 8. | AVT (Entecavir &/or tenofovir) - The number of patients being on dual therapy in AVT arm was 3. | Oral | NA |
| Serrano-Villar et al. 2021 (5) | Oral | Induction with 10 capsules (single dose), followed by weekly maintenance FMT with five capsules for 7 weeks. In total, each subject received 45 capsules, implying the delivery of 30 g of stools over 8 weeks. | Stable ART | Stable ART + Placebo capsules containing glycerol, cocoa butter, and inert, non-toxic brown pigment (in place of stool) | Oral | Induction with 10 placebo capsules (single dose), followed by weekly maintenance with five capsules for 7 weeks. |
| Boicean et al. 2022 (6) | Colonoscopy | Single administration after the first 10 days of antibiotic treatment using 50 g of donated feces (less than 6 h after defecation) in 500 mL of 0.9% saline, mixing to obtain a homogeneous solution, and filtering. Patients ceased antibiotic treatment 24 h prior to the instillation procedure. | Vancomycin: 45 (97.82%)  Both vancomycin and metronidazole: 1 (2.17%) | Vancomycin: 11 (27.5%)  Both vancomycin and metronidazole: 29 (72.5%) | Vancomycin (oral) & Metronidazole (I.V) | Vancomycin (250 mg, administered four times a day) & Metronidazole (500 mg, administered three times a day if needed after the initial 10 days of Vancomycin treatment) |
| Ren et al. 2017 (7) | Gastroscope, with the bacterial suspension being injected into the third part of the duodenum | At least 30 g of isolated bacterial precipitation was collected and resuspended in normal saline at a final volume of 80 mL. FMT was performed every 4 weeks. | Entecavir or tenofovir disoproxil fumarate | Entecavir or tenofovir disoproxil fumarate | Oral | NA |
| Vujkovic-Cvijin et al. 2017 (8) | Colonoscopy | Participants underwent standard bowel purge (Golytely) the day preceding FMT and the 250 mL stool suspension was introduced via colonoscopy and delivered into the ileum, cecum, and ascending colon | ART | ART | NA | NA |

Abbreviations: NG: Nasogastric; ART: Antiretroviral therapy; AVT: Antiviral therapy; NA: Not available; FMT: fecal microbiota transplantation;

*: patients who received antibiotics treatment 5 days prior to FMT

**References**

1. SahBandar IN, Chew GM, Corley MJ, Pang APS, Tsai N, Hanks N, et al. Changes in gastrointestinal microbial communities influence HIV-specific CD8+ T-cell responsiveness to immune checkpoint blockade. Aids. 2020;34(10):1451-60.

2. Utay NS, Monczor AN, Somasunderam A, Lupo S, Jiang ZD, Alexander AS, et al. Evaluation of Six Weekly Oral Fecal Microbiota Transplants in People with HIV. Pathog Immun. 2020;5(1):364-81.

3. Karolewska-Bochenek K, Lazowska-Przeorek I, Grzesiowski P, Dziekiewicz M, Dembinski L, Albrecht P, et al. Faecal Microbiota Transfer - a new concept for treating cytomegalovirus colitis in children with ulcerative colitis. Ann Agric Environ Med. 2021;28(1):56-60.

4. Chauhan A, Kumar R, Sharma S, Mahanta M, Vayuuru SK, Nayak B, et al. Fecal Microbiota Transplantation in Hepatitis B e Antigen-Positive Chronic Hepatitis B Patients: A Pilot Study. Dig Dis Sci. 2021;66(3):873-80.

5. Serrano-Villar S, Talavera-Rodríguez A, Gosalbes MJ, Madrid N, Pérez-Molina JA, Elliott RJ, et al. Fecal microbiota transplantation in HIV: A pilot placebo-controlled study. Nat Commun. 2021;12(1):1139.

6. Boicean A, Neamtu B, Birsan S, Batar F, Tanasescu C, Dura H, et al. Fecal Microbiota Transplantation in Patients Co-Infected with SARS-CoV2 and Clostridioides difficile. Biomedicines. 2022;11(1).

7. Ren YD, Ye ZS, Yang LZ, Jin LX, Wei WJ, Deng YY, et al. Fecal microbiota transplantation induces hepatitis B virus e-antigen (HBeAg) clearance in patients with positive HBeAg after long-term antiviral therapy. Hepatology. 2017;65(5):1765-8.

8. Vujkovic-Cvijin I, Rutishauser RL, Pao M, Hunt PW, Lynch SV, McCune JM, et al. Limited engraftment of donor microbiome via one-time fecal microbial transplantation in treated HIV-infected individuals. Gut Microbes. 2017;8(5):440-50.
