# Supplementary material for: Safety and efficacy of fecal microbiota transplantation for viral diseases: A systematic review of clinical trials
Source: PLoS One. 2024 Oct 21;19(10):e0311731. doi: 10.1371/journal.pone.0311731 (PMC11493255; doi:10.1371/journal.pone.0311731)
Supplement: S3 Table — (DOCX) [file pone.0311731.s003.docx]

# S3 Table. Quality assessment of uncontrolled studies.

| Study ID | 1 | 2 | 3 | 4 | 5 | 6 | 7 | 8 | 9 | Overall scores |
| --- | --- | --- | --- | --- | --- | --- | --- | --- | --- | --- |
| SahBandar et al. 2020 (1) | Yes | Yes | Not available | Yes | Yes | Yes | Yes | Yes | Yes | 8 |
| Utay et al. 2020 (2) | Yes | Yes | Not available | Yes | Yes | Yes | Yes | Yes | Yes | 8 |
| Karolewska-Bochenek et al. 2020 (3) | Yes | Yes | Yes | Yes | Yes | Yes | Yes | No | Yes | 8 |

1. Was the study question or objective clearly stated?

2. Was the study population clearly and fully described, including a case definition?

3. Were the cases consecutive?

4. Were the subjects comparable?

5. Was the intervention clearly described?

6. Were the outcome measures clearly defined, valid, reliable, and implemented consistently across all study participants?

7. Was the length of follow-up adequate?

8. Were the statistical methods well-described?

9. Were the results well-described?

Overall quality rating: Good: 7-9; Fair: 4-6; and Poor: 0-3

**References**

1. SahBandar IN, Chew GM, Corley MJ, Pang APS, Tsai N, Hanks N, et al. Changes in gastrointestinal microbial communities influence HIV-specific CD8+ T-cell responsiveness to immune checkpoint blockade. Aids. 2020;34(10):1451-60.

2. Utay NS, Monczor AN, Somasunderam A, Lupo S, Jiang ZD, Alexander AS, et al. Evaluation of Six Weekly Oral Fecal Microbiota Transplants in People with HIV. Pathog Immun. 2020;5(1):364-81.

3. Karolewska-Bochenek K, Lazowska-Przeorek I, Grzesiowski P, Dziekiewicz M, Dembinski L, Albrecht P, et al. Faecal Microbiota Transfer - a new concept for treating cytomegalovirus colitis in children with ulcerative colitis. Ann Agric Environ Med. 2021;28(1):56-60.
