# Supplementary material for: Safety and efficacy of fecal microbiota transplantation for viral diseases: A systematic review of clinical trials
Source: PLoS One. 2024 Oct 21;19(10):e0311731. doi: 10.1371/journal.pone.0311731 (PMC11493255; doi:10.1371/journal.pone.0311731)
Supplement: S4 Table — (DOCX) [file pone.0311731.s004.docx]

# S4 Table. Quality assessment of interventional controlled studies.

| Study ID | 1 | 2 | 3 | 4 | 5 | 6 | 7 | 8 | 9 | 10 | 11 | 12 | 13 | 14 | Overall scores |
| --- | --- | --- | --- | --- | --- | --- | --- | --- | --- | --- | --- | --- | --- | --- | --- |
| Chauhan et al.  2020 (1) | No | No | Not available | Not available | Not available | Yes | Yes | Yes | Yes | Yes | Yes | No | Yes | No | 7 |
| Serrano-Villar et al.  2021 (2) | Yes | Yes | Yes | Yes | Yes | Yes | Yes | Yes | Yes | Yes | Yes | No | Yes | Yes | 13 |
| Boicean et al.  2022 (3) | No | No | Not available | Not available | Not available | Yes | Yes | Yes | Yes | Yes | Yes | No | Yes | No | 7 |
| Ren et al.  2017 (4) | No | No | No | No | Yes | Yes | Yes | No | Yes | Yes | Yes | No | Yes | Yes | 8 |
| Vujkovic-Cvijin et al.  2017 (5) | No | No | No | No | Not available | Yes | Yes | Yes | Yes | Yes | Yes | No | Yes | No | 7 |

1. Was the study described as randomized, a randomized trial, a randomized clinical trial, or an RCT?

2. Was the method of randomization adequate (i.e., use of randomly generated assignment)?

3. Was the treatment allocation concealed (so that assignments could not be predicted)?

4. Were study participants and providers blinded to treatment group assignment?

5. Were the people assessing the outcomes blinded to the participants' group assignments?

6. Were the groups similar at baseline on important characteristics that could affect outcomes (e.g., demographics, risk factors, co-morbid conditions)?

7. Was the overall drop-out rate from the study at endpoint 20% or lower of the number allocated to treatment?

8. Was the differential drop-out rate (between treatment groups) at endpoint 15 percentage points or lower?

9. Was there high adherence to the intervention protocols for each treatment group?

10. Were other interventions avoided or similar in the groups (e.g., similar background treatments)?

11. Were outcomes assessed using valid and reliable measures, implemented consistently across all study participants?

12. Did the authors report that the sample size was sufficiently large to be able to detect a difference in the main outcome between groups with at least 80% power?

13. Were outcomes reported or subgroups analyzed prespecified (i.e., identified before analyses were conducted)?

14. Were all randomized participants analyzed in the group to which they were originally assigned, i.e., did they use an intention-to-treat analysis?

Overall quality rating: Good: 11-14; Fair: 7-10; and Poor: 0-6

**References**

1. Chauhan A, Kumar R, Sharma S, Mahanta M, Vayuuru SK, Nayak B, et al. Fecal Microbiota Transplantation in Hepatitis B e Antigen-Positive Chronic Hepatitis B Patients: A Pilot Study. Dig Dis Sci. 2021;66(3):873-80.

2. Serrano-Villar S, Talavera-Rodríguez A, Gosalbes MJ, Madrid N, Pérez-Molina JA, Elliott RJ, et al. Fecal microbiota transplantation in HIV: A pilot placebo-controlled study. Nat Commun. 2021;12(1):1139.

3. Boicean A, Neamtu B, Birsan S, Batar F, Tanasescu C, Dura H, et al. Fecal Microbiota Transplantation in Patients Co-Infected with SARS-CoV2 and Clostridioides difficile. Biomedicines. 2022;11(1).

4. Ren YD, Ye ZS, Yang LZ, Jin LX, Wei WJ, Deng YY, et al. Fecal microbiota transplantation induces hepatitis B virus e-antigen (HBeAg) clearance in patients with positive HBeAg after long-term antiviral therapy. Hepatology. 2017;65(5):1765-8.

5. Vujkovic-Cvijin I, Rutishauser RL, Pao M, Hunt PW, Lynch SV, McCune JM, et al. Limited engraftment of donor microbiome via one-time fecal microbial transplantation in treated HIV-infected individuals. Gut Microbes. 2017;8(5):440-50.
